# Uterine Cancer Mortality in White and African American Females in Southeastern North Carolina

**DOI:** 10.1155/2020/6734031

**Published:** 2020-09-30

**Authors:** Julia Kravchenko, Igor Akushevich, Sung Han Rhew, Pankaj Agarwal, H. Kim Lyerly

**Affiliations:** ^1^Environmental Health Scholars Program, Division of Surgical Sciences, Department of Surgery, Duke University School of Medicine, Durham, NC, USA; ^2^The Biodemography of Aging Research Unit, Social Science Research Institute, Duke University, Durham, NC, USA; ^3^Memory Keepers Medical Discovery Team, University of Minnesota Medical School, Duluth, MN, USA; ^4^Department of Pathology, Duke University School of Medicine, Durham, NC, USA

## Abstract

The residents of southeastern North Carolina (NC) are exposed to multiple socioeconomic and environmental risk factors and have higher mortality rates for a number of diseases. Uterine cancer mortality is known to vary dramatically by race, so we analyzed uterine cancer mortality in populations defined by zip codes in this area to investigate the contributions of various environmental risk factors to race-specific disease patterns. *Methods*. Zip code specific mortality and hospital admissions for uterine cancer from 2007 to 2013 were analyzed using the NC State Center for Health Statistics data and the Inpatient Database of the Healthcare Cost and Utilization Project datafiles, respectively. Results were adjusted for age, income, education, health insurance coverage, prevalence of current smokers, and density of primary care providers. *Results*. Uterine cancer mortality rates were generally higher in African American (32.5/100,000, 95% CI = 18.9–46.1) compared to White (19.6/100,000, 95% CI = 12.3–26.9) females. Odds ratios (ORs) of uterine cancer death were higher in White females (OR = 2.27, *p* < 0.0001) residing within zip codes with hog concentrated animal feeding operations (CAFOs) (hog density >215 hogs/km^2^) than in White females residing in non-CAFO communities. African American females living near CAFOs had less pronounced increase of uterine cancer death (OR = 1.08, *p*=0.7657). *Conclusion*. White females living in adjacent to hog CAFOs areas of southeastern NC have lower rates of mortality from uterine cancer than African American females, but they have higher odds of death compared to their counterparts living in other NC areas. African American females living near CAFOs also have modest increases from their high baseline mortality. While the observed associations do not prove a causation, improving access to screening and medical care is important to mitigate this health issues in southeastern NC.

## 1. Introduction

The elevated mortality from uterine cancer in southeastern North Carolina (NC) compared to the overall United States (US) average is striking: 23.1% increase in mortality rates from 1999 to 2014 in NC vs. 14.6% in the US [1]. Previous studies have demonstrated that socioeconomic characteristics, behavioral factors, differences in access to treatment, and variability in treatment approaches contribute to disparities in uterine cancer [2], but other factors such as rural residence, income, or location of surgery departments do not [3, 4]. Southeastern NC is unique; in that, it contains multiple large hog concentrated animal feeding operations (CAFOs) [5]; therefore, the impact of this specific environmental factor should be included in analysis of health conditions in this area. Previous studies have focused on the occupational health risks of CAFOs and have confirmed an increased risk of respiratory and infectious diseases among workers [6–8]. Studies among the residents of adjacent to hog CAFOs communities showed a higher risk of bacterial infections, arterial hypertension, depression, and respiratory and neurological disorders [7, 9–13]. In addition, these communities also been reported to have higher total and infant mortality rates and increased risks of kidney disease, anemia, and septicemia [14].

Elevated concentrations of endocrine-disrupting chemicals (EDCs) have been detected in swine manure [15–18], and elevated levels of estrogens were detected in waters surrounding hog CAFOs [19–22]. While epidemiological studies have reported relationships between EDCs and endometrial cancer [23–27], the specific associations with uterine cancer risk among females living in close proximity to hog CAFOs remain understudied. It remains unclear whether and to what extent higher mortality from uterine cancer in the southeastern NC is associated with the additional impact of hog CAFOs, i.e., beyond the disparities associated with socioeconomic characteristics, access to medical care, and other potential risk factors. The objective of our study was to evaluate race-specific patterns of uterine cancer mortality and hospital admission among African American and White females in southeastern NC. We sought to identify how demographic, socioeconomic, behavioral factors, access to medical care, and living in close proximity to hog CAFOs impact uterine cancer specific mortality and hospitalization.

## 2. Materials and Methods

Data on uterine cancer mortality for 2007–2013 were obtained from publicly available data sources at the NC State Center for Health Statistics (SCHS), Vital Statistics Death, UNC Dataverse [28–34]. Data on hospital admissions were obtained from the Inpatient Database of the Healthcare Cost and Utilization Project (HCUP SID data) [35]. Malignant neoplasms of body of uterus (i.e., corpus uteri) were analyzed using International Classification of Diseases- (ICD-) 9 code 182 and ICD-10 code C54. Information on median household income and education level was obtained from the 2010–2014 American Community Survey for each NC zip code (a ZIP code is an acronym for zone improvement plan, i.e., a postal code used by the US Postal Service): median household income was scaled by $10,000, and education level was defined as a percentage of people aged 25+ who attained a bachelor's degree or higher educational level. Information on the availability of primary care providers (per 100,000 population) and the percentage of uninsured individuals in each of NC counties (an administrative subdivision of a state that consists of a geographic region with specific boundaries; county is a larger administrative unit than a zip code) were obtained from the Area Health Resources Files (AHRF) of the Health Resources and Service Administration (HRSA) for 2008 and 2010–2013. Data on prevalence of current smokers in age-specific groups were obtained on a county level from the Behavioral Risk Factor Surveillance System (BRFSS, CDC) for 2008–2013. Information on hog operations registered in NC was obtained from the Division of Water Resources (DWR), the North Carolina Department of Environmental Quality (NC DEQ). Animal operations are defined by the General Statute 143–215.10B as feedlots involving 250 or more swine with a liquid waste management system.

**Figure 1:**
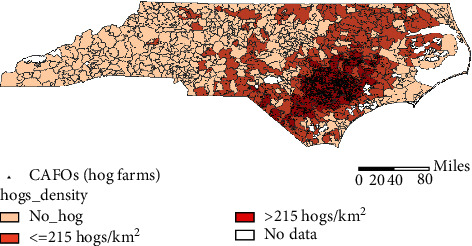
Locations of NC zip codes from the study and control groups^#^ (^#^NC zip codes with the density of hogs >215 hogs/km^2^ and NC zip codes without registered at the DWR hog farms, respectively. Additionally, NC zip codes with the density of hogs ≤215 hogs/km^2^ are shown. Black dots represent locations of hog farms registered at the DWR).

*Methods*. Analysis of health outcomes was performed at the geographic level of ZIP Code Tabulation Areas (abbreviated in the text to zip code). Methodologically, this study is nested in a larger study analyzing total and disease-specific morbidity and mortality in populations living in the southeastern NC [14]. The study group included 56 southeastern NC zip codes with the upper quartile of hog density in NC (>215 hogs/km^2^); it totals in 1,393,824 person-years. The control group included 601 NC zip codes that had none of the hog farms registered at the DWR; it totals in 25,476,730 person-years. Geographic distribution of zip codes of the study group and the control group is shown in Figure 1. All analyses were performed for uterine cancer as an underlying cause among females aged 65+, i.e., age group in which over 67% of uterine cancer deaths in the US occur [36, 37].

### 2.1. Age-Adjusted Rates of Mortality and Hospital Admissions

Age-adjusted rates (per 100,000) of mortality and hospital admissions were calculated in the study and control groups. The US Census 2010 population counts were used for the rates calculation. The 95% confidence intervals (CIs) were estimated based on approximation by Keyfitz [38].

### 2.2. Logistic Regression Analyses

Logistic regression analysis adjusted for age, median household income, education, health insurance coverage, number of primary care providers, and current smokers prevalence was used to evaluate whether a percentage of deaths due to uterine cancer (as an underlying cause among all-cause deaths occurred during the study period) was higher in the study group than in the control group. Analysis was also performed for hospital admissions for uterine cancer (as the percentage among all-cause hospital admissions during the study period). This approach allowed avoiding likely uncertainties in population counts in NC zip codes over the study period, thus resulting in reduction of potential bias. The control group was a referent group for calculating the odds ratios (ORs). For multiple comparisons, the Bonferroni correction was applied. SAS Proc Logistic (SAS 9.4 statistical package; SAS Institute, Cary, NC) was used to evaluate the ORs, 95% CIs, and *p* values.

### 2.3. The Distance from the Source of Potential Contamination: The “DiSC” Analysis

We used the Distance from the Source of potential Contamination (DiSC) analysis to investigate how the odds of death and hospital admission change with closer proximity to hog CAFO. This approach was used in our previous studies [14]. This method assumes that the concentration of contaminants has a maximum at a hog CAFO's location and decreases with remoteness from CAFO according to a two-dimensional normal (i.e., “bell-shaped”) distribution (justified by the theory of diffusion from a point source [39]). Since there are no direct measurements allowing for estimation of the radius of potential impacts of associated with hog CAFO contaminants, radius-specific analyses were performed at 2 km, 5 km, 10 km, and 20 km (as described in the study by Kravchenko et al. [14]). The values obtained in this analysis were further incorporated into logistic regression approach described in Subsection 2.2 of the Methods section.

### 2.4. Comparing Uterine Cancer Outcomes in Matched NC Zip Codes

The greedy matching algorithm [40] was used for propensity score-based matching of zip codes (this approach is described in detail in the study by Kravchenko et al. [14]). Zip codes from the control group were matched to zip codes from the study group to form the matched group A and the matched group B. The matched group A included 56 zip codes from the control group matched by the percentage of African Americans, children aged 0–19, individuals aged 65+, and the median household income. The matched group B included 55 zip codes from the control group that were matched using educational level (i.e., by percentage of people with bachelor's or higher degree) additionally to the variables used in the matched group A. Characteristics of both matched groups A and B are shown in .Supplemental Table S2. The age-adjusted rates of uterine cancer mortality and hospital admissions were calculated.

### 2.5. Sensitivity Analysis

Uterine malignancies have been analyzed using additional codes to include the unspecified part of malignant neoplasm of uterus: ICD-9 codes 179 and 182, and ICD-10 codes C54 and C55 (further termed as “unspecified + body of uterus” cancer in the Supplemental Tables S3–S7). Uterine cancer was analyzed when being the underlying and secondary cause of death and primary and secondary diagnoses of hospital admission. For potential impact of living in rural/urban areas, zip codes of the cities of Charlotte and Raleigh were excluded to account for predominantly rural locations of hog CAFOs in NC. Furthermore, 18 urbanized areas (defined by the US Census Bureau as having 50,000 or more residents) were excluded from the analysis. Analysis was performed for younger females aged 45–64 years. The method of generalized estimating equation (GEE) was used (SAS Proc Genmod) to account for possible correlations between uterine cancer records in specific NC zip codes.

### 2.6. Ethics Statement

Data analysis was designed and performed according to ethical standards of the responsible committee on human studies and the Helsinki Declaration (1975, revised in 1983) and have been approved by the University Health System Institutional Review Board (IRB).

## 3. Results

### 3.1. Demographic and Socioeconomic Characteristics

There was a higher percentage of African American females living in NC zip codes with >215 hogs/km^2^ (the study group) than in the control group: 32.7% vs. 20.0% (*p* < 0.001), respectively (Table 1). Median household income, educational degree, and the rate of primary care providers were lower, and the prevalence of current smokers was higher in the study group. Person-years of observations in race-specific groups of females are shown in Supplemental Table S1.

### 3.2. Age-Adjusted Rates of Mortality and Hospital Admission

African American females had higher age-adjusted rates of uterine cancer mortality than White females in both the study and the control groups (Table 2). White females had higher mortality (19.6/100000, 95% CI = 12.3–26.9) in the study compared to the control (9.7/100000, 95% CI = 8.6–10.8), while for African American females, between-the-group difference did not reach the statistical significance (32.5/100000, 95% CI = 18.9–46.1 in the study vs. 24.4/100000, 95% CI = 20.1–28.7 in the control group). In the study group, both African American and White females had higher rates of hospital admissions for uterine cancer, with the rates for African American exceeding the rates for White females (Table 2).

### 3.3. Logistic Regression Analysis

When adjusted for age, White females had increased OR of uterine cancer death in the study group, and these ORs remained significantly increased after adjustment for cofactors (OR = 2.27, *p* < 0.0001) (Table 3). While the death ORs for African American females were also increased after adjustment for cofactors, these ORs did not reach the statistical significance (OR = 1.08, *p*=0.7657). Race-specific patterns of hospital admissions for uterine cancer opposed the patterns observed for mortality; after adjustment for cofactors, hospital admission ORs were significantly increased for African American (OR = 1.89, *p*=0.0013) but not for White females (OR = 1.03, *p*=0.8158).

### 3.4. The Results of the DiSC Analysis

For both, White and African American females in the study group, ORs of uterine cancer death were increasing with closer proximity to hog CAFO (Table 4). For White females, this pattern remained significant after adjustment for cofactors: the highest OR was observed within 2 km from hog CAFO (OR = 1.65, *p*=0.0014) and then gradually decreased with increasing distance to CAFO (OR = 1.22, *p*=0.0005, at 5 km; OR = 1.11, *p*=0.0002, at 10 km; and OR = 1.07, *p*=0.0002, at 20 km to CAFO). For African American females, death ORs were below the statistical significance: OR = 1.38 at 2 km (*p*=0.1075), OR = 1.09 at 5 km (*p*=0.2229), OR = 1.04 at 10 km (*p*=0.3217), and OR = 1.02 at 20 km (*p*=0.3821).

### 3.5. The Results of Analysis in Matched Zip Codes

White females in the study group had higher rates of uterine cancer mortality (19.6/100000, 95% CI = 12.4–26.9) than White females in the matched group A (11.6/100000, 95% CI = 6.8–16.5) and matched group B (9.5/100000, 95% CI = 4.5–14.4) (Table 5). They also had higher rates of hospital admission for uterine cancer in the study group compared to both matched groups. For African American females, mortality rates in the study group did not significantly exceed the rates in the matched groups, but hospital admission rates were significantly higher in the study group compared to the matched groups.

### 3.6. Sensitivity Analysis

The results of analyses of “unspecified + body of uterus” cancer and uterine cancer as a multiple cause of death and hospital admission have confirmed race-specific patterns of age-adjusted rates obtained in the main analysis (Supplemental Table S3), ORs (Supplemental Tables S4 and S5), and age-adjusted rates in the study group compared to the matched groups (Supplemental Table S6). Sensitivity analysis with exclusion of urban/urbanized areas (Supplemental Table S7) in general confirmed the findings of the main analysis with one exclusion, i.e., the rates of hospital admissions for uterine cancer were slightly lower in rural areas for both White and African American females. At age 45–64 years, there was a tendency for higher rates of uterine cancer mortality in the study group compared to the control group among both White and African American females; however, between-the-group difference in mortality rates was statistically significant only for cancer of corpus uteri as a multiple cause of death in White females (Supplemental Table S7). The results of GEE analysis (Supplemental Table S8) were in agreement with the results of the main analysis.

## 4. Discussion

The higher odds of uterine cancer mortality in White females living in the southeastern NC in zip codes with hog CAFOs compared to White women from other areas of NC (i.e., without hog CAFOs) is a new finding in our study. This mortality risk persisted after adjustment for age, median household income, education, health insurance coverage, numbers of primary care providers, and prevalence of current smokers and was increasing with a closer proximity to hog CAFO, thus providing further support about the possible role of the hog CAFOs in uterine cancer outcomes. However, while White females living near hog CAFOs had higher death ORs, African American females living in this NC area had higher age-adjusted mortality rates from uterine cancer compared to White females. This finding is in agreement with other studies that suggested the role of diagnosis at advanced stages, poorer treatment choice, longer wait times from diagnosis to definitive surgery, lower adherence to treatment [2, 4, 36, 41], a higher estrogen exposure and obesity prevalence [42, 43], and a higher prevalence of aggressive histologic tumor subtypes and low-grade tumors [44] in African American females. While the increase in uterine cancer death ORs did not reach the statistical significance in African American females living near hog CAFOs in the southeastern NC (compared to African American females living in NC areas without hog CAFOs), further studies in larger African American population are needed to investigate whether their generally higher rates of uterine cancer mortality are associated with proximity to hog CAFOs in a manner similar to White females or their increased rates are determined by other causes.

The finding on substantially increased ORs of uterine cancer death observed in White females living in closer proximity to hog CAFOs could be explained, at least partially, by potential occupational settings. According to the Decennial Census Occupation Report for NC [45], there were 79.5% of White females and 11.3% of African American females employed in a farming sector in Duplin County and 89.3% and 10.7% of females, respectively, employed in a farming in Sampson County. These counties have the highest number of hogs in hog CAFOs in NC. Therefore, while no specific details of the exact farming occupation were available in Census data [45] for that period, it is plausible that hog farming is the main type of a farming-related employment in these counties. It could be speculated that a fraction of population of White females employed in hog farming in southeastern NC is higher than of African American females; therefore, they have a higher probability of occupational exposures. Moreover, because the workers of hog CAFOs likely live close to the farm, they are likely to be exposed residentially additionally to their occupational exposure.

While the results of our study do not prove individual causal associations with potential exposures to contaminants from hog CAFOs, they clearly demonstrate specific patterns of uterine cancer mortality in the southeastern NC, including increasing mortality with a closer proximity to the CAFO. The impact of CAFO on health has been previously reported, including reports of an increased risk of lung disease in hog farm workers [6, 46–50] and a higher risk of diarrhea, respiratory symptoms, neurological disorders, depression, fatigue, arterial hypertension kidney disease, septicemia, anemia, tuberculosis, and higher infant and total mortality among the residents of communities located near hog CAFOs [7, 10–14, 51–57]. While no previous reports evaluated the risk of uterine cancer in either occupational exposures or in communities located in close proximity to hog farms, there is a plausible connection due to the high concentration of EDC surrounding CAFOs. Livestock are reported as a major source of EDCs contamination in the environment [58, 59] as large quantities of estriol, 17-estradiol, bisphenol A, and 17-ethinyloestradiol have been identified in cow dung and chicken and duck manure, with the highest concentrations being detected in swine manure [15, 16, 20, 60–62]. Furthermore, hog waste is minimally treated [17, 18], and it is often spread from the lagoons to agricultural land as a source of the nutrients for crops. This can lead to contamination of soil, surface water, and groundwater with EDCs [19–22]. While recent techniques were proposed for substantially decreasing estrogens levels in livestock manure [63, 64], more available techniques are still on a high demand.

Estrogen is the important risk factor of uterine cancer. Type I uterine cancer (an endometrioid carcinoma that represents 80%–90% of uterine neoplasms) is associated with an exposure to estrogens [65–68]. Obtaining a firm epidemiological demonstration of EDCs-related carcinogenesis is difficult because of multiple cofactors that could impact uterine cancer risk; however, such studies gradually became more frequent [23]. Estrogens can affect reproductive functions in humans and act as mitogens in estrogen receptor- (ER-) positive cells, having the potential to promote DNA instability, cellular hyperplasia, and neoplastic transformation into carcinoma [24, 25]. Potential mechanisms of action of EDCs could include epigenomic changes [26, 27] and impact on carcinogen metabolism, immune system, oxidation, and inflammation [23]. Even low-dose exposures to EDCs have been shown to impact the health, and such low-dose effects cannot be directly calculated from the effects of high-dose exposures [23].

Lower rates of hospital admissions could also contribute to a lower survival among White females with uterine cancer. Studies showed that disadvantaged populations in the southeastern NC are exposed to socioeconomic factors associated with higher disease rates and have lower access to medical care compared to other NC areas [69, 70], especially in the communities located near hog CAFOs [71, 72].

*Methodological aspects*, *study limitations*, *and future perspectives*. At present, no existing datasets provide individual-level information (that is necessary for ideal research scenario for such study) on multiple cofactors in relatively large size populations. Therefore, for this study, the data on individual-level health outcomes were combined with the area-based data on socioeconomic, behavioral, and environmental factors. This limitation is often reported for these types of analyses. However, public health specialists and policymakers began gradually shifting from the analysis of individual-level health outcomes toward community and population group level studies. The motivation behind the increasing interest to more general than individual-level environmental data analysis is that this approach allows to obtain information that can be directly used for optimization of access to medical care in the communities at their large, thus making them attractive targets for public health interventions bearing a greater impact [73].

Due to combination of unique features of the southeastern NC and specific set of coexisting and interrelating risk factors in this area and due to the differences in access to medical care across the US states, the results of our study cannot be directly applied to other US states with hog CAFOs (e.g., Iowa, Minnesota, and some other states). However, the developed analytic algorithm allows for using this type of approach for analysis of factors that impact health outcomes in disadvantaged populations, which are specific for each state.

Information on tumor characteristics (i.e., tumor stage or histologic type) and cancer treatment was not available from the datasets we used. Further studies based on the Surveillance, Epidemiology, and End Results (SEER) linked to Medicare data and datafiles of the regional tumor registries can be used to obtain these variables. Also, further detailed analysis could include an individual history of occupational exposure in agriculture including the hog farming sector and individual information on exact locations of the residence in the areas adjacent to hog CAFOs when this information will be available. Further studies can also include factors such as wind direction, temperature, humidity, severe rainfalls with flood danger, and other factors related to weather and climate characteristics in southeastern NC that could impact potential distributions of contaminants in air and water. However, we expect that weather-related factors impact predominantly acute diseases or diseases with acute attacks/decompensations (e.g., asthma, stroke, and certain infections) rather than they will substantially affect mortality from uterine cancer.

The results of this study provide justification for further detailed surveillance on socioeconomic and behavioral risk factors and on access to cancer diagnosis and treatment in southeastern NC communities adjacent to hog CAFOs. This study provides a rationale for survey for specific diseases such as uterine cancer and potential environmental exposures in such communities. Furthermore, community-based studies can include direct measurements of the levels of organic and nonorganic contaminants in the air, water, soil, and human tissue samples, with specific focus on estrogen compounds and subsequent analysis of possible correlations between such exposures and uterine cancer incidence, tumor characteristics, access to the treatment, and patients' survival in race-specific population groups. Information obtained will be useful for planning the strategies of early diagnosis of uterine cancer and improving access and adherence to treatment in the southeastern NC communities, especially those residing within 5 km from hog CAFO.

## Figures and Tables

**Table 1:** Demographic and socioeconomic characteristics of the study group and control group^#^, 2007–2013.

Characteristics	Study group	Control group
Race (%)		
White	57.6%^*∗∗*^	73.1%
African American	32.7%^*∗∗*^	20.0%
American Indian	4.2%^*∗∗*^	0.8%
Asian	0.4%^*∗∗*^	2.5%
Others	5.3%^*∗∗*^	3.5%

Age structure of female population (%)		
0–9 years old	13.0%^*∗∗*^	12.4%
10–19 years old	13.5%^*∗*^	12.8%
20–44 years old	31.1%	33.8%
45–64 years old	26.6%	26.4%
65+ years old	15.9%	14.6%

Median household income (the U.S. dollars)	$36520^*∗∗*^	$46414
Bachelor or higher degree education (%)	11.1%^*∗∗*^	23.5%
Number of primary care providers (per 100,000 population)	51^*∗∗*^	77
Percentage of uninsured individuals	18.5%	17.8%
Smokers prevalence among those aged 24+ years old (%)	25.9%^*∗∗*^	24.0%

^*∗*^ *p* < 0.05 compared to the control group; ^*∗∗*^*p* < 0.001 compared to the control group. ^#^NC zip codes with the density of hogs >215 hogs/km^2^ and NC zip codes without registered at the DWR hog farms, respectively.

**Table 2:** Uterine cancer in the study and control groups^#^: mortality and hospital admission rates, 2007–2013.

Outcome	Race	Study group	Control group
Death rate	African American	32.5^a^ (18.9–46.1)^b^	24.4 (20.1–28.7)
	White	19.6 (12.3–26.9)	9.7 (8.6–10.8)

Hospital admissions rate	African American	63.5 (44.3–82.7)	40.9 (35.4–46.5)
	White	44.1 (33.2–55.0)	34.0 (31.9–36.1)

^a^Rates are calculated per 100000; ^#^NC zip codes with the density of hogs >215 hogs/km^2^ and NC zip codes without registered at the DWR hog farms, respectively; ^b^95% CIs are shown in the parentheses.

**Table 3:** Death and hospital admission odds ratios^a^ (ORs) of females with uterine cancer, 2007–2013.

Outcome	Type of adjustment	Race	OR	*p* value
Death	Age-adjusted	African American	1.25	0.3321
		White	1.71^#^	0.0069
	Multivariable^b^	African American	1.08	0.7657
		White	2.27^#^	<0.0001

Hospital admissions	Age-adjusted	African American	1.85^#^	0.0003
		White	1.03	0.8154
	Multivariable	African American	1.89^#^	0.0013
		White	1.03	0.8158

^a^Control group was a referent group; analysis is adjusted by age, income, education, health insurance, prevalence of current smokers, and number of primary care providers; ^#^remains significant under Bonferroni correction for multiple comparisons.

**Table 4:** DiSC^a^ analysis: death ORs within different distances from hog CAFOs in NC; multivariable analysis^b^, 2007–2013.

Race	Distance from the CAFO			
	2 km	5 km	10 km	20 km
White	1.65^#^, *p*=0.0014	1.22^#^, *p*=0.0005	1.11^#^, *p*=0.0002	1.07^#^, *p*=0.0002
African American	1.38, *p*=0.1075	1.09, *p*=0.2229	1.04, *p*=0.3217	1.02, *p*=0.3821

^a^Distance from the source of potential contamination; ^b^analysis adjusted by age, income, education, health insurance, prevalence of current smokers, and availability of primary care providers; ^#^remains significant under Bonferroni correction for multiple comparisons.

**Table 5:** Analysis in the study and matched groups A and B^#^: uterine cancer mortality and hospital admission rates^a^, 2007–2013.

Outcome	Race	Study group	Matched group A	Matched group B
Mortality	White	19.6 (12.4–26.9)^b^	11.6 (6.8–16.5)	9.5 (4.5–14.4)
	African American	32.5 (18.9–46.1)	26.2 (15.0–37.5)	36.8 (21.8–51.8)

Hospital admissions	White	44.1 (33.2–55.0)	34.5 (25.9–43.1)	30.3 (21.1–39.4)
	African American	63.5 (44.3–82.7)	41.8 (27.8–55.9)	35.3 (20.6–50.1)

^#^Southeastern NC zip codes with >215 hogs/km^2^ (the study group) and NC zip codes without hog CAFOs matched by demographic characteristics such as the percentage of African Americans and percentage of children and adults aged 65+ in population, and median household income (matched group A) and additionally matched by the percentage of the residents aged 25+ with bachelor's or higher degree (matched group B); ^a^age-adjusted rates (per 100000) among females aged 65+; ^b^95% CIs are shown in the parentheses.

## Data Availability

Data on hospital admissions in NC are not publicly available; these data have been obtained from the Healthcare Cost and Utilization Project (HCUP) in accordance with their procedure of data request. “As we stated in original text of our manuscript, these data can be obtained under the DUA” at the Agency for Healthcare Research and Quality at www.hcup-us.ahrq.gov/sidoverview.jsp.

## Supplementary Materials

Supplementary Materials Table S1: person-years of observations in race-specific groups of female residents of the southeastern NC zip codes with >215 hogs/km^2^ (the study group) and NC zip codes without hog CAFOs (the control group), 2007–2013. Table S2: characteristics used for matching zip codes in the matched group A (matched by demographic characteristics such as percentage of African American and percentage of children and adults aged 65+ in population and median household income) and the matched group B (additionally to the matched group A matched by percentage of the residents aged 25+ with bachelor's or higher degree) to the study group, North Carolina, 2007–2013 (the results are presented as mean ± standard error). Table S3: age-adjusted rates (per 100000) of mortality and hospital admissions among females with uterine cancer living in the southeastern NC zip codes with >215 hogs/km^2^ (the study group) and NC zip codes without hog CAFOs (the control group), 2007–2013 (95% CIs are shown in the parentheses). Table S4: odds ratios (ORs) of death and hospital admissions among females with uterine cancer living in the southeastern NC zip codes with >215 hogs/km^2^ (the study group): age-adjusted and multivariable analysis adjusted by six cofactors (age, income, education, health insurance, prevalence of current smokers, and number of primary care providers), North Carolina, 2007–2013 (the control group is a referent group.). Table S5: the Distance from the Source of potential Contamination (“DiSC”) analysis: death ORs of White and African American females having residences in the southeastern NC zip codes within different distances from hog CAFOs; multivariable analysis (adjusted by age, income, education, health insurance, prevalence of current smokers, and availability of primary care providers), 2007–2013. Table S6: age-adjusted rates (per 100000) of mortality and hospital admissions among females with uterine cancer living in the southeastern NC zip codes with >215^2^ hogs/km^2^ (the study group) and in NC zip codes without hog CAFOs matched by demographic characteristics (percentage of African American and percentage of children and adults aged 65+ in population) and median household income (the matched group A) and additionally matched by percentage of the residents aged 25+ with bachelor's or higher degree (the matched group B), 2007–2013 (95% confidence intervals are shown in parentheses). Table S7: the results of exclusion of urban/urbanized areas and analysis for age group 45–64 years old: mortality and hospital admissions rates (per 100000) of uterine cancer in the southeastern NC zip codes with >215 hogs/km^2^ (the study group) and NC zip codes without hog CAFOs (the control group), 2007–2013 (95% confidence intervals are shown in parentheses). Table S8: the results of generalized estimating equation (GEE) analysis: death and hospital admission ORs in African American and White females living in the southeastern NC zip codes with >215 hogs/km^2^ (the study group): uterine cancer as the underlying cause of death/primary diagnosis and multiple cause of death/hospital admission, 2007–2013 (the control group is a referent group).
