# Supplementary material for: Uterine Cancer Mortality in White and African American Females in Southeastern North Carolina
Source: J Environ Public Health. 2020 Sep 30;2020:6734031. doi: 10.1155/2020/6734031 (PMC7545445; doi:10.1155/2020/6734031)
Supplement: Supplementary Materials — Table S1: person-years of observations in race-specific groups of female residents of the southeastern NC zip codes with >215 hogs/km2 (the study group) and NC zip codes without hog CAFOs (the control group), 2007–2013. Table S2: characteristics used for matching zip codes in the matched group A (matched by demographic characteristics such as percentage of African American and percentage of children and adults aged 65+ in population and median household income) and the matched group B (additionally to the matched group A matched by percentage of the residents aged 25+ with bachelor's or higher degree) to the study group, North Carolina, 2007–2013 (the results are presented as mean ± standard error). Table S3: age-adjusted rates (per 100000) of mortality and hospital admissions among females with uterine cancer living in the southeastern NC zip codes with >215 hogs/km2 (the study group) and NC zip codes without hog CAFOs (the control group), 2007–2013 (95% CIs are shown in the parentheses). Table S4: odds ratios (ORs) of death and hospital admissions among females with uterine cancer living in the southeastern NC zip codes with >215 hogs/km2 (the study group): age-adjusted and multivariable analysis adjusted by six cofactors (age, income, education, health insurance, prevalence of current smokers, and number of primary care providers), North Carolina, 2007–2013 (the control group is a referent group.). Table S5: the Distance from the Source of potential Contamination (“DiSC”) analysis: death ORs of White and African American females having residences in the southeastern NC zip codes within different distances from hog CAFOs; multivariable analysis (adjusted by age, income, education, health insurance, prevalence of current smokers, and availability of primary care providers), 2007–2013. Table S6: age-adjusted rates (per 100000) of mortality and hospital admissions among females with uterine cancer living in the southeastern NC zip codes with >2152 hogs/km2 (the study [file 6734031.f1.docx]

**Supplemental Tables S1-S8.**

**Table S1.** Person-years of observations in race-specific groups of female residents of southeastern NC zip codes with >215 hogs/km^2^ (the Study group) and NC zip codes without hog CAFOs (the Control group), 2007-2013.

| **Demographic characteristics** | **Study group** | **Control group** |
| --- | --- | --- |
| Total female population | 1393824 | 25476730 |
| Race:  White | 802148 | 18631305 |
| African-American | 455801 | 5097331 |
| American Indian | 57783 | 207002 |
| Asian | 4853 | 642125 |
| Other | 73239 | 900070 |
| Age structure:  0-9 years old | 181725 | 3162451 |
| 10-19 years old | 187568 | 3270313 |
| 20-44 years old | 432731 | 8608495 |
| 45-64 years old | 370072 | 6727144 |
| 65+ years old | 221728 | 3708327 |

**Table S2**. Characteristics used for matching zip codes in Matched group A (matched by demographic characteristics such as percent of African-Americans, and percent of children and adults aged 65+ in population, and median household income) and Matched group B (additionally to Matched group A matched by percent of the residents aged 25+ with bachelor’s or higher degree) to the Study group, North Carolina, 2007-2013. (The results are presented as Mean ± Standard error).

| **Characteristics** | **Matched**  **group A** | **Study group** | **Matched**  **group B** | **Study group** |
| --- | --- | --- | --- | --- |
| % of African-Americans | 30.9^1^±3.0% | 30.2±1.8% | 24.8±3.0% | 30.2±1.8% |
| % of children (0-19 years old) | 25.8^1^±0.8% | 26.2±0.4% | 24.6±0.6% | 26.2±0.4% |
| % of adults (65+ years) | 15.4±0.9% | 16.2±0.5% | 18.0±0.7% | 16.2±0.5% |
| Median household income ($US) | $35910±$1086 | $36520±$919 | $34891±$1009 | $36520±$919 |
| % of people with bachelor or higher degree education among aged 25+ | n/a^a^ | n/a | 9.2±0.7% | 11.1±0.5% |

Notes: ^a^non-applicable; ^1^the percent showed in this table are for total population in respective zip codes (i.e., not for female population only as the percent presented in Table 1).

**Table S3**. Age-adjusted rates (per 100000) of mortality and hospital admissions among females with uterine cancer living in southeastern NC zip codes with >215 hogs/km^2^ (the Study group) and NC zip codes without hog CAFOs (the Control group), 2007-2013. (95% CIs are shown in the parentheses).

| **Outcome** | **Uterine cancer** | **Race** | **Underlying cause of death/primary diagnosis** | | | **Multiple cause of death/**  **hospital admission^1^** | |  |
| --- | --- | --- | --- | --- | --- | --- | --- | --- |
|  |  |  | **Study group** | **Control group** | **Study group** | | **Control group** | |
| Death | Corpus uteri | AA^2^ | 32.5 (18.9-46.1) | 24.4 (20.1-28.7) | 37.1 (22.5-51.6) | | 27.3 (22.8-31.9) | |
|  |  | White | **19.6**^3^ (12.3-26.9) | 9.7 (8.6-10.8) | **21.5** (13.9-29.0) | | 11.1 (9.9-12.2) | |
|  | Unspecified+  body of uterus | AA | 61.6 (42.7-80.5) | 46.7 (40.8-52.7) | 69.9 (49.9-89.9) | | 51.7 (45.5-58.0) | |
|  |  | White | **30.3** (21.2-39.3) | 18.0 (16.5-19.5) | **34.7** (25.1-44.3) | | 21.8 (20.2-23.4) | |
| Hospital admissions | Corpus uteri | AA | **63.5** (44.3-82.7) | 40.9 (35.4-46.5) | **136.3** (108.2-164.5) | | 82.6 (74.7-90.5) | |
|  |  | White | 44.1 (33.2-55.0) | 34.0 (31.9-36.1) | **67.6** (54.2-81.1) | | 51.0 (48.5-53.6) | |
|  | Unspecified+  body of uterus | AA | 69.0 (49.1-89.0) | 51.6 (45.4-57.9) | **170.7** (139.3-202.2) | | 116.9 (107.5-126.3) | |
|  |  | White | **44.8** (33.8-55.8) | 37.7 (35.5-39.9) | **80.2** (65.6-94.9) | | 62.5 (59.7-65.3) | |

Note: ^1^counted out from twelve diagnoses in medical records for each death or hospital admission; ^2^African-American; ^3^**bold font** means significant difference in rates when compared to respective Control group.

**Table S4.** Odds ratios (ORs) of death and hospital admissions among females with uterine cancer living in southeastern NC zip codes with >215 hogs/km^2^ (the Study group): age-adjusted and multivariable analysis adjusted by six co-factors (age, income, education, health insurance, smoking prevalence, and number of primary care providers), North Carolina, 2007-2013. (Control group is a referent group.)

| **Outcome** | **Cancer** | **Race, type of analysis** | **Underlying cause of death/primary diagnosis** | **Multiple cause of death/**  **hospital admission^1^** |
| --- | --- | --- | --- | --- |
| Death | Corpus uteri | AA^2^, age-adjusted | 1.25, p=0.3321 | 1.27, p=0.2730 |
|  |  | White, age-adjusted | 1.71^#^, p=0.0069 | **1.66**^3, #^**,** p=0.0072 |
|  |  | AA, multivariable | 1.08, p=0.7657 | 1.00, p=0.9937 |
|  |  | White, multivariable | **2.27**^#^, p<0.0001 | **2.12**^#^**,** p=0.0002 |
|  | Unspecified+  body of uterus | AA, age-adjusted | 1.22, p=0.2540 | 1.25, p=0.1592 |
|  |  | White, age-adjusted | **1.40,** p=0.0337 | **1.35,** p=0.0405 |
|  |  | AA, multivariable | 1.29, p=0.2003 | 1.25, p=0.2383 |
|  |  | White, multivariable | **1.84**^#^**,** p=0.0003 | **1.68**^#^**,** p=0.0008 |
| Hospital admissions | Corpus uteri | AA, age-adjusted | **1.85**^#^, p=0.0003 | **1.93**^#^**,** p<0.0001 |
|  |  | White, age-adjusted | 1.03, p=0.8154 | 1.06, p=0.6003 |
|  |  | AA, multivariable | **1.89**^#^, p=0.0013 | **2.11**^#^**,** p<0.0001 |
|  |  | White, multivariable | 1.03, p=0.8158 | 1.16, p=0.1704 |
|  | Unspecified+  body of uterus | AA, age-adjusted | **1.59**^#^**,** p=0.0033 | **1.71**^#^**,** p<0.0001 |
|  |  | White, age-adjusted | 0.96, p=0.7406 | 1.03, p=0.7507 |
|  |  | AA, multivariable | **1.62**^#^**,** p=0.0092 | **1.94**^#^**,** p<0.0001 |
|  |  | White, multivariable | 0.98, p=0.8598 | 1.15, p=0.1708 |

Notes: ^1^counted out from twelve diagnoses in medical records for each death or hospital admission; ^2^African-American; ^3^**bold font** means significant ORs values; ^#^remains significant under Bonferroni correction for multiple comparisons.

**Table S5**. The **Di**stance from the **S**ource of potential **C**ontamination (“DiSC”) analysis: death ORs of White and African-American females having residences in southeastern NC zip codes within different distances from hog CAFOs, multivariable analysis (adjusted by age, income, education, health insurance, smoking, and availability of primary care providers), 2007-2013.

| **Uterine cancer site** | **Race** | **Underlying cause of death** | | | | **Multiple cause of death^1^** | | | |
| --- | --- | --- | --- | --- | --- | --- | --- | --- | --- |
|  |  | **2 km** | **5 km** | **10 km** | **20 km** | **2 km** | **5 km** | **10 km** | **20 km** |
| Corpus uteri | White | **1.65**^3, #^  p=0.0014 | **1.22**^#^,  p=0.0005 | **1.11**^#^  p=0.0002 | **1.07**^#^  p=0.0002 | **1.60**^#^  p=0.0018 | **1.20**^#^  p=0.0008 | **1.10**^#^  p=0.0003 | **1.06**^#^**,**  p=0.0003 |
|  | AA^2^ | 1.38  p=0.1075 | 1.09  p=0.2229 | 1.04  p=0.3217 | 1.02  p=0.3821 | 1.25  p=0.2543 | 1.06  p=0.4510 | 1.02  p=0.5368 | 1.01  p=0.5167 |
| Unspecified  +body of uterus | White | **1.51**^#^  p=0.0012 | **1.18**^#^  p=0.0005 | **1.09**^#^  p=0.0002 | **1.05**^#^  p<0.0001 | **1.42**^#^  p=0.0032 | **1.15**^#^  p=0.0016 | **1.08**^#^  p=0.0006 | **1.05**^#^  p=0.0004 |
|  | AA | **1.38**  p=0.0328 | 1.10  p=0.0712 | 1.05  p=0.0931 | 1.03  p=0.0551 | **1.35**  p=0.0372 | 1.09  p=0.0972 | 1.04  p=0.1166 | 1.03  p=0.0641 |

Notes: ^1^counted out from twelve diagnoses in medical records for each death or hospital admission; ^2^African-American; ^3^**bold font** means significant ORs values; ^#^remains significant under Bonferroni correction for multiple comparisons.

**Table S6.** Age-adjusted rates (per 100000) of mortality and hospital admissions among females with uterine living in southeastern NC zip codes with >215 hogs/km^2^ (the Study group) and in NC zip codes without hog CAFOs matched by demographic characteristics (percent of African-Americans, and percent of children and adults aged 65+ in population) and median household income (Matched group A) and additionally matched by percent of the residents aged 25+ with bachelor’s or higher degree (Matched group B), 2007-2013. (95% confidence intervals are shown in parentheses).

| **Outcome** | **Cancer** | **Race** | **Underlying cause of death/primary diagnosis** | | | **Multiple cause of death/hospital admission^1^** | | |
| --- | --- | --- | --- | --- | --- | --- | --- | --- |
|  |  |  | **Study**  **group** | **Matched**  **group A** | **Matched**  **group B** | **Study**  **group** | **Matched**  **group A** | **Matched**  **group B** |
| Mortality | Corpus uteri | White | 19.6  (12.4-26.9) | **11.6**^3^  (6.8-16.5) | **9.5**  (4.5-14.4) | 21.5  (13.8-29.0) | **13.9**  (8.6-19.3) | **10.7**  (5.5-16.0) |
|  |  | AA | 32.5  (18.9-46.1) | 26.2  (15.0-37.5) | 36.8  (21.8-51.8) | 37.1  (22.6-51.6) | 28.8  (17.0-40.5) | 43.1  (26.9-59.4) |
|  | Unspecified+  body of uterus | White | 30.3  (21.2-39.3) | 21.7  (15.1-28.2) | **15.5**  (9.2-21.8) | 34.7  (25.1-44.3) | **24.7**  (17.7-31.7) | **18.7**  (11.8-25.7) |
|  |  | AA | 61.6  (42.8-80.5) | 53.4  (37.4-69.4) | 53.3  (35.4-71.2) | 69.9  (49.9-89.9) | 59.5  (42.6-76.3) | 60.9  (41.8-80.1) |
| Hospital admissions | Corpus uteri | White | 44.1  (33.2-55.0) | 34.5  (25.9-43.1) | **30.3**  (21.1-39.4) | 67.6  (54.2-81.1) | 60.0  (48.7-71.4) | **47.0**  (35.6-58.4) |
|  |  | AA | 63.5  (44.3-82.7) | **41.8**  (27.8-55.9) | **35.3**  (20.6-50.1) | 136.3  (108.2-164.5) | **94.4**  (73.2-115.7) | **94.7**  (70.5-118.9) |
|  | Unspecified+  body of uterus | White | 44.8  (33.8-55.8) | 36.2  (27.4-45.0) | 35.3  (25.4-45.2) | 80.2  (65.6-94.9) | 71.0  (58.7-83.3) | **61.8**  (48.7-74.8) |
|  |  | AA | 69.0  (49.1-89.0) | 51.4  (35.8-66.9) | **44.8**  (28.2-61.5) | 170.7  (139.3-202.2) | **125.0**  (100.6-149.4) | **120.4**  (93.2-147.7) |

Notes: ^1^counted out from twelve diagnoses in medical records for each death or hospital admission; ^2^African-American; ^3^**bold font** means significant difference in rates when compared to respective Study group.

**Table S7.** The results of exclusion of urban/urbanized areas, and analysis for age group 45-64 years old: mortality and hospital admissions rates (per 100000) of uterine cancer in southeastern NC zip codes with >215 hogs/km^2^ (the Study group) and NC zip codes without hog CAFOs (the Control group), 2007-2013. (95% confidence intervals are shown in parentheses).

| **Outcome** | **Cancer** | **Race** | | | | **Underlying cause of death/primary diagnosis** | | | **Multiple cause of death/hospital admission^1^** | | | | |
| --- | --- | --- | --- | --- | --- | --- | --- | --- | --- | --- | --- | --- | --- |
|  |  |  |  |  |  | **Study group** | **Control group** | | **Study group** | | | **Control group** | |
| **After excluding Charlotte and Raleigh from analysis** | | | | | | | | | | | | | |
| Death | Corpus uteri | | | AA^2^ | 32.5 (8.9-46.1) | | | 26.9 (21.7-32.0) | 37.1 (22.6-51.6) | | 30.1 (24.7-35.5) | | |
|  |  |  |  | White | **19.6**^3^ (12.4-26.9) | | | 9.6 (8.4-10.7) | **21.5** (13.9-29.0) | | 11.0 (9.8-12.2) | | |
|  | Unspecified+  body of uterus | | | AA | 61.6 (42.8-80.5) | | | 47.4 (40.7-54.2) | 69.9 (49.9-89.9) | | 52.8 (45.7-60.0) | | |
|  |  |  |  | White | **30.3** (21.2-39.3) | | | 17.4 (15.9-19.0)) | **34.7** (25.1-44.3) | | 21.3 (19.6-23.0) | | |
| Hospital admissions | Corpus uteri | | | AA | **63.5** (44.3-82.7) | | | 41.9 (35.5-48.2) | **136.3** (108.2-164.5) | | 85.3 (76.2-94.4) | | |
|  |  |  |  | White | 44.1 (33.2-55.0) | | | 33.4 (31.3-35.6) | **67.6** (54.2-81.1) | | 49.5 (46.8-52.1) | | |
|  | Unspecified+  body of uterus | | | AA | 69.0 (49.1-89.0) | | | 50.3 (43.3-57.2) | **170.7** (139.3-202.2) | | 114.1 (103.6-124.6) | | |
|  |  |  |  | White | 44.8 (33.8-55.8) | | | 36.5 (34.2-38.8) | **80.2** (65.6-94.9) | | 59.7 (56.8-62.6) | | |
| **After excluding 18 urbanized areas from analysis** | | | | | | | | | | | | | |
| Death | Corpus uteri | | AA | | 35.4 (20.3-50.6) | | | 27.7 (20.6-34.8) | 40.7 (24.4-56.9) | | | 32.8 (25.1-40.5) | |
|  |  |  | White | | **20.8** (12.9-28.6) | | | 9.7 (8.3-11.0) | **22.8** (14.6-31.0) | | | 11.1 (9.7-12.5) | |
|  | Unspecified+  body of uterus | | AA | | 63.4 (43.0-83.8) | | | 48.8 (39.4-58.1) | 72.9 (51.1-94.7) | | | 57.1 (47.0-67.2) | |
|  |  |  | White | | **31.7** (22.0-41.4) | | | 17.7 (15.9-19.5) | **36.6** (26.2-46.9) | | | 21.7 (19.8-23.7) | |
| Hospital admissions | Corpus uteri | | AA | | **63.7** (43.2-84.3) | | | 43.8 (34.9-52.6) | **138.9** (108.4-169.3) | | | 82.8 (70.6-95.1) | |
|  |  |  | White | | 41.5 (30.4-52.5) | | | 32.0 (29.5-34.4) | **65.8** (51.9-79.7) | | | 46.4 (43.5-49.4) | |
|  | Unspecified+  body of uterus | | AA | | 70.0 (48.6-91.4) | | | 52.3 (42.6-62.1) | **174.6** (140.6-208.7) | | | 106.9 (93.0-120.9) | |
|  |  |  | White | | 42.2 (31.1-53.4) | | | 35.3 (32.8-37.9) | **75.8** (60.9-90.8) | | | 57.2 (54.0-60.5) | |
| **Uterine cancer outcomes in females aged 45-64 years old** | | | | | | | | | | | | | |
| Death | Corpus uteri | | AA | | | 4.5 (0.9-8.2) | | 3.9 (2.8-4.9) | | 5.2 (1.4-9.1) | | | 4.0 (2.9-5.1) |
|  |  |  | White | | | 4.5 (1.7-7.2) | | 2.4 (2.0-2.8) | | **5.2** (2.3-8.2) | | | 2.6 (2.2-3.0) |
|  | Unspecified+  body of uterus | | AA | | | 11.1 (5.5-16.8) | | 9.1 (7.5-10.8) | | 11.8 (6.0-17.6) | | | 9.8 (8.1-11.5) |
|  |  |  | White | | | 5.7 (2.6-8.9) | | 4.9 (4.3-5.5) | | 6.5 (3.2-9.8) | | | 5.3 (4.7-5.9) |

Note: ^1^counted out from twelve diagnoses in medical records for each death or hospital admission; ^2^African-American; ^3^**bold font** means significant difference in rates compared to the Control group.

**Table S8**. The results of generalized estimating equation (GEE) analysis: death and hospital admission ORs in African-American and White females living in southeastern NC zip codes with >215 hogs/km^2^ (the Study group): uterine cancer as underlying cause of death/primary diagnosis and multiple cause of death/hospital admission, 2007-2013. (The Control group is a referent group)

| **Race** | **Outcome** | **Underlying cause of death/primary diagnosis** | **Multiple cause of death/hospital admission^1^** | |
| --- | --- | --- | --- | --- |
| African-American | Deaths | 1.18, p=0.3154 | | 1.23, p=0.1852 |
|  | Hospital admissions | **1.51**^2^, p=0.0158 | | **1.59**^#^, p=0.0091 |
| White | Deaths | **1.54**^#^, p=0.0035 | | **1.48**^#^, p=0.0058 |
|  | Hospital admissions | 1.03, p=0.8317 | | 1.11, p=0.4175 |

Note: ^1^counted out from twelve diagnoses in medical records for each death or hospital admission; ^2^**bold font** means significant OR value; ^#^remains significant under Bonferroni correction for multiple comparisons.
